# In reply: frequency distribution of age at breast cancer diagnosis provides complementary clinical insight

**DOI:** 10.1093/oncolo/oyag295

**Published:** 2026-07-31

**Authors:** Anna N Wilkinson, Carmina Ng, Larry F Ellison, Jean M Seely

**Affiliations:** Department of Family Medicine, University of Ottawa, Ottawa, ON K1G 5Z3, Canada; Centre for Population Health Data, Statistics Canada, Government of Canada, Ottawa, ON K1A 0T6, Canada; Centre for Population Health Data, Statistics Canada, Government of Canada, Ottawa, ON K1A 0T6, Canada; Department of Radiology, University of Ottawa, Ottawa, ON K1H 8L6, Canada

We thank the corresponding author for her interest in our article and for her praise of its contribution to the literature. We appreciate her thoughtful critique of our presentation of peak age at diagnosis as one of the elements describing breast cancer patterns among race/ethnicity groups in Canada, and we welcome the opportunity to clarify the purpose of this analysis in our paper.^1^ We agree that age-standardization is indispensable for comparing overall incidence, or other metrics, across populations with different underlying age structures. Multiple age-specific comparisons of incidence rates across populations are also very useful in providing information on a more refined level. In addition, we think that an analysis of the actual age distribution of observed breast cancer cases can be complementarily informative in the clinical setting.

In Table 1 of our original paper, we presented age-standardized incidence rates to inform on potential summary differences in breast cancer risk across race/ethnicity in Canada. These rates provide an age-adjusted summary of overall risk for each race/ethnicity in the hypothetical scenario where all groups had the same age structure. Age-specific incidence rates were presented in Table 2 of our paper to examine breast cancer risk across race/ethnicity on a more granular level. Our presentation of thesmoothed age distribution of cases, including the peak age at diagnosis, was not intended as an additional measure of age-specific risk. Rather, it was meant to provide an indication of the age at which the percentage of cases diagnosed was highest in the Canadian population for each group (ie, the age at which disease volume was concentrated within a given subpopulation). These results are essential in providing clinically interpretable information that can aid in the development and evaluation of interventions, such as when the use of diagnostic or screening tests may be recommended. They can also lead to an appreciation of the need for increased awareness among health care professionals of the potential for breast cancer in a population so that patients’ symptoms and concerns are not inappropriately dismissed. The frequency at which patients within their respective populations present themselves to health care professionals is also important given the nuances of differing aggressive molecular subtypes and mortality according to race/ethnicity groups. Finally, these results are important for comparability with other similar studies that also report age distribution of cases.^2^^,^^3^

Differences in the actual case distribution across racial/ethnic groups may arise from differing population age structures across these groups in Canada, but they may also arise from differences in age-specific risk across groups. Were the age distributions to be used as another comparison of the age risk profile of breast cancer diagnosis across racial/ethnic groups, we agree that some form of age-adjustment may be appropriate. The corresponding author has suggested one way in which this could be accomplished, while another has been previously published.^4^ However, we caution that when the reference population is known to be older than the studied populations, as in this circumstance, the estimated peak/median values are sensitive to the choice of reference population.^5^

No single metric can fully characterize complex relationships, and so the best practice is to present multiple complementary measurements as we have done. Taken together, these descriptors can provide critical information allowing race/ethnicity considerations to be appropriately incorporated into clinical applications.
